# Notch mRNA Expression in Drosophila Embryos Is Negatively Regulated at the Level of mRNA 3′ Processing

**DOI:** 10.1371/journal.pone.0008063

**Published:** 2009-11-30

**Authors:** Andrew K. Shepherd, Ravinder Singh, Cedric S. Wesley

**Affiliations:** 1 Department of Microbiology and Molecular Genetics, University of Vermont, Burlington, Vermont, United States of America; 2 Department of Molecular, Cell and Developmental Biology, University of Colorado, Boulder, Colorado, United States of America; Centre de Regulació Genòmica, Spain

## Abstract

Notch receptor regulates differentiation of almost all tissues and organs during animal development. Many mechanisms function at the protein level to finely regulate Notch activity. Here we provide evidence for Notch regulation at an earlier step - mRNA 3′ processing. Processing at the Notch consensus polyadenylation site appears by default to be suppressed in Drosophila embryos. Interference with this suppression, by a mutation, results in increased levels of polyadenylated Notch mRNA, excess Notch signaling, and severe developmental defects. We propose that Notch mRNA 3′ processing is negatively regulated to limit the production of Notch protein and render it a controlling factor in the generation of Notch signaling.

## Introduction

Notch (N) signaling specifies binary cell fates and refines morphological patterns during differentiation of almost all tissues or organs in animals. N, a cell surface receptor, and Delta, a cell surface anchored ligand, mediate N signaling. N and Delta binding results in the release of the N intracellular domain (N^intra^) from the cell surface. N^intra^ translocates to the nucleus and activates transcription of target genes. Cells that suppress N signaling commit to one developmental fate whereas cells that activate N signaling commit to the alternative developmental fate [1]–[9]. N signaling is very finely and tightly regulated. A mere 1.5–2X difference in gene dosage, or very low levels of constitutive activation, results in mutant phenotypes [10]–[20]. A number of mechanisms function at the level of N protein modification, trafficking, recycling, and degradation to regulate N activity [5]–[6], [21]–[26]. Whether N activity is regulated at the level of mRNA as well is uncertain.

Genetic screens indicate that many RNA binding proteins play important roles in N signaling [27]–[28]. We neither know their Notch pathway targets nor the mechanisms employed except for *mushashi*, which represses the translation of the *numb* mRNA [29]). Many of these RNA binding proteins are part of the basic mRNA 3′ processing machinery such as *hiiragi* that encodes a Poly(A) Polymerase in *Drosophila melanogaster*. Basic mRNA 3′ processing factors are required for processing of all poly(A) tailed mRNA but they show special interaction with N signaling, to the extent of even reproducing N mutant phenotypes [30]–[31]. It is not known why N signaling is particularly sensitive to changes in levels of basic mRNA 3′ processing factors and which among the more than 60 N signaling pathway genes is the target. Here we present evidence that the *N* gene itself is a target. N mRNA 3′ processing at the consensus poly(A) site appears to be usually suppressed in Drosophila embryos. A mutation in this poly(A) site increases the production of polyadenylated N mRNA, N^intra^, and N signaling in association with severe developmental defects. Default suppression of mRNA 3′ processing at the N consensus poly(A) site might be important for limiting the production of the N protein, thereby enabling sensitive responses to developmental cues.

## Materials and Methods

### Fly Procedures

Wild type (*y w*), *FM7a* balancer, and DSC *N^nd1^* stocks were obtained from the Drosophila Stock Center. *N^nd1^/C(1)A/Y* stock was obtained from Dr. Spyros Artavanis-Tsakonas (Harvard University). Standard Drosophila techniques [32] were used for generating iso-chromosomal lines and processing embryos for immuno-staining, northern blotting, and western blotting procedures. Embryos were staged according to reference [33].

*N^nd1-dse^* is a temperature sensitive allele [34]. *N^nd1-dse^* embryos are more or less wild type at the permissive temperature of 18°C and mutant at the restrictive temperature of 29°C. Embryonic mortality is about 60% at the restrictive temperature. *N^nd1-dse^* and the wild type *y, w* embryos were collected at 18°C for 2–5 hours, aged to desired stages (taking into account the slower developmental rate at this temperature), and then shifted to 29°C for desired time periods before processing them for molecular procedures. *N^nd1-dse^* and the wild type embryos were processed in parallel and in an identical manner. Embryos used for immuno-staining were transferred to the restrictive temperature half an hour before N signaling is used to specify neuronal and epidermal precursor cells (after about six hours of embryogenesis at 18°C).

### Molecular Procedures

Procedures followed for DNA extraction, cloning, cDNA synthesis, immuno-staining, northern blotting, western blotting, and collection of staged embryos are described [18], [22], [25], [35], [and 36]. Northern blotting was used to check the levels of N and rp49 mRNA before proceeding with RT-PCR based analyses. For western blots, embryos were pulverized in 1X Laemmli buffer with β-marcaptoethanol and protease inhibitors. These blots were probed with N intracellular (NI; [18]) or hsp 70 antibodies (Sigma). Immuno-staining of embryos was performed with an anti-Hunchback antibody (Paul Macdonald) and signals developed with a HRP conjugated secondary antibody.

Primers used in PCR analysis for the isolation of the original *N^nd-1^* allele were: 5′ primer 1 (5′cggcggaggaggaggtggtggtggtggtgttgg3′); downstream 3′ primer 1 (5′aatcatccagatcacggtca3′); and deletion 3′ primer (5′ttcaggtccaagcccgctg3′). Primers flanking the deletion that were used for confirming the absence of the deletion in the original *N^nd-1^* allele were: 5′ primer 1 (5′cggcggaggaggaggtggtggtggtggtgttgg3′) and 3′ primer 2 (5′tatcgagggcggattcatttg3′). Sequencing was performed at the UVM Vermont Cancer Center Core Facility. PAT assays were done following the procedures described [37] [and 38]. The N specific primer used in these assays was 5 primer 2 (5′cacaaaaatcaccaatggaaacgtataagtc3′) and the rp49 specific primer used was 5′agtatctgatgcccaacatcg3′. Unprocessed (extended) N transcript analysis was done using 5′ primer 2 (5′cacaaaaatcaccaatggaaacgtataagtc3′) as the 5′ primer and 3′ primer 3, (5′cgggtttgtgtgtgtgtgtc3′) as the 3′ primer. Total RNA in the samples was assessed using rp49 primers 5′agtatctgatgcccaacatcg3′ and 5′ ttccgaccaggttacaagaac3′. High fidelity pfu turbo enzyme (Stratagene) was used for all PCR reactions. Megascript kit (Ambion) and poly(dT) 3′ primer with a 3-fold degenerate (A/G/C) 3′ end (for site of mRNA cleavage assay), poly(dT) 3′ primer (for PAT assay), or random hexamers (for extended transcript assay) were used to prepare cDNA. For northern blotting, PAT, and PCR assays embryos were collected 60–90 minutes after the flies were shifted to the restrictive temperature; for western blotting assay, embryos were collected after 120 minutes at the restrictive temperature.

For making actin promoter-GFPcoding-N3′UTR+DSE and actin promoter-GFPcoding-N^nd1^3′UTR+ N^nd1^ DSE constructs, the KpnI-NotI GFP coding sequence fragment from pEGFP (Clontech) was inserted after the ∼2.7 kb EcoRI fragment containing actin 5C promoter in the pBluescript (pBS) plasmid. N3′UTR+N DSE and N^nd1^3′UTR+ N^nd1^ DSE sequence were amplified with primers containing NotI sites, cloned into the NotI site at the end of the GFP coding sequence in pEGFP. N^nd1^3′UTR+N DSE was generated in a similar manner using a primer including the wild type DSE sequence and the N^nd1^3′UTR+ N^nd1^ DSE template. Plasmids with the correct orientation and sequence were determined by sequencing and used to transiently transfect S2 cells. DNA was extracted from these cells and used to transfect bacteria for assessing transfection efficiency based on the number of bacterial colony forming units. We found the transfection efficiency to be the similar with different constructs (data not shown).

Images and figures were processed using Photoshop (Adobe) and Canvas (Deneba) programs. Any adjustments were applied to whole images.

## Results

### The Original *N^nd-1^* Allele Contains a Mutation in the Consensus Poly(A) Site of the *N* Gene

The original *N^nd-1^* mutation was mapped downstream of the DNA encoding Ankyrin repeats in the intracellular domain of the N protein [19, 34, 39; http://flybase.org). Initial reports that the *N^nd-1^* protein coding sequence downstream of Ankyrin repeats contains two amino acid polymorphisms were shown to be erroneous by later studies (http://flybase.org). All reports before the year 2000 showed that the coding sequence in the *N^nd-1^* allele is complete indicating that the *N^nd-1^* lesion lies in the 3′ UTR and the adjacent sequence important for mRNA 3′ processing (http://flybase.org). In 2007, Harding-Theobald et al. [40] reported that the *N^nd-1^* stock in the Drosophila Stock Center (DSC) contains a 41 base pair deletion within the coding region resulting in a frame-shift that would replace the terminal 129 amino acids of the N protein with a 63 amino acid-long novel sequence (http://flybase.org). Thus, the Harding-Theobald et al. (2007) report suggested the accumulation of a second mutation in the *N^nd-1^* allele.

**Figure 1:**
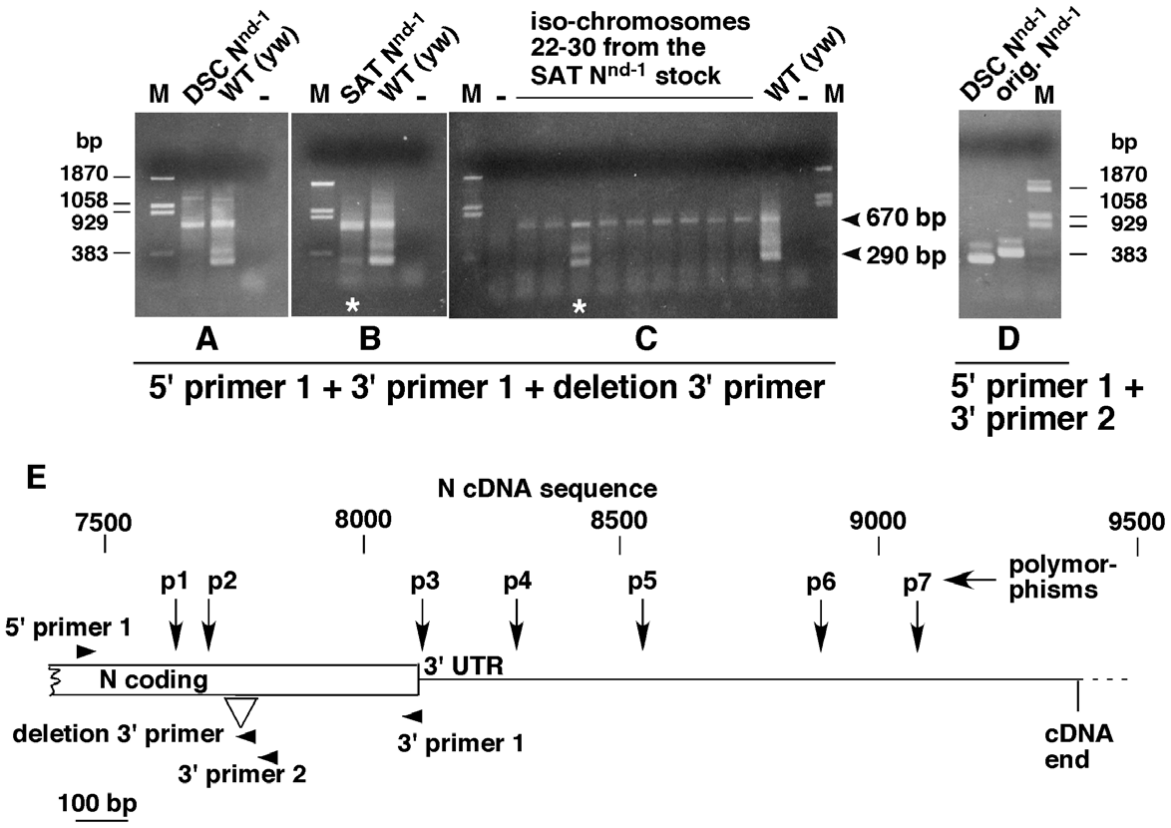
The original *N^nd-1^* allele is present in the SAT *N^nd-1^* stock. (**A**) DSC *N^nd-1^* stock is homozygous for the Harding-Theobald et al. [40] deletion, as the deletion 3′ primer does not yield the 290 bp PCR product. (**B**) SAT *N^nd-1^* stock contains the original N^nd1^ allele, as the 290 bp PCR product is present at a low level (lane with white asterisk). (**C**) In a sub-sample of 9 iso-chromosomal lines isolated from the SAT *N^nd-1^* stock one is revealed as the original *N^nd-1^* chromosome (lane with white asterisk). Photographs of ethidium bromide stained agarose gels are shown. PCR included one 5′ N primer (5′ primer 1) and two 3′ N primers; one located downstream of the deletion (3′ primer 1 that yields the 670 bp product) and one inside the deletion (deletion 3′ primer that yields the 290 bp product from alleles without the Theobald-Harding et al. deletion). (**D**) PCR primers flanking the Theobald-Harding et al. deletion (5′ primer 1 and 3′ primer 2) yield PCR products of different sizes indicating that this deletion is present in the DSC *N^nd-1^* allele but not in the original *N^nd-1^* allele. A 307 bp product was expected with the Theobald-Harding et al. deletion (from the DSC *N^nd-1^* allele) and a 348 bp product was expected without this deletion (from the original *N^nd-1^* allele). -  =  no template control; M  =  marker DNA. (**E**) Schematic representation of the Theobald-Harding et al., deletion (∇), primers used determine its presence or absence in fly lines (arrow heads), and polymorphisms (↓p1 – p5) detected in the study (p1  =  CAA to CAG in the eighth Glutamine codon in the opa region; p2  =  CAA to CAG in the last Glutamine codon in the opa region; p3  =  G to C at position 8161; p4 =  a T deletion at position 8300; and p5 =  a T insertion at position 8544. Coding region polymorphisms p1 and p2 do not change the amino acid sequence, confirming the earlier report by others that there is no change in the amino acid sequence of the original nd^1^ allele (FlyBase).

To confirm our inference we obtained a culture of the *N^nd-1^* stock from Dr. Spyros Artavanis-Tsakonas (SAT) who was the source for the DSC *N^nd-1^* stock. We performed PCR analysis on the DSC and SAT *N^nd-1^* flies with a 5′ primer upstream of the deletion reported by Harding-Theobald et al. (2007) and two 3′ primers: one downstream of the deletion and one within the deleted sequence. From the DNA of wild-type flies we expected a 670 base pair (bp) product with the downstream 3′ primer 1 and a 290 bp product with the deletion 3′primer. The 290 bp-product was not expected from the DNA of flies carrying the deletion. Results showed that the DSC *N^nd-1^* fly stock does not yield the 290 bp product confirming the presence of the deletion (**Fig. 1A**). There was no evidence that the original *N^nd-1^* allele was present in this stock as increasing the number of PCR cycles, the amount of template, or the number of flies used for DNA extraction (up to 100) did not yield the 290 bp product. We always obtained the expected products from both the wild type and the DSC *N^nd-1^* DNA using PCR primers located outside the deletion (data not shown).

The same assay on the SAT *N^nd-1^* fly stock showed that this stock contains the original *N^nd-1^* allele at a low frequency as we obtained a low level of the 290 bp PCR product (**Fig. 1B**, lane with asterisk). To isolate this allele from the SAT *N^nd-1^* stock, we established 47 iso-chromosomal lines and assayed them for the deletion. **Figure 1C** shows a portion of our result in which one out of nine iso-chromosomes carries the original *N^nd-1^* allele indicated by the presence of the 290-bp product. The band above the 290 bp product is single-strand DNA produced by an imbalance in the activities of the primers (data not shown). Further experiments using primers that flanked the deletion confirmed that the original *N^nd-1^* chromosome does not contain the deletion, as PCR product obtained from this chromosome was longer than the product obtained from the DSC *N^nd1^* chromosome (**Fig. 1D**). Overall, we isolated six original *N^nd-1^* chromosomes.

**Figure 2:**
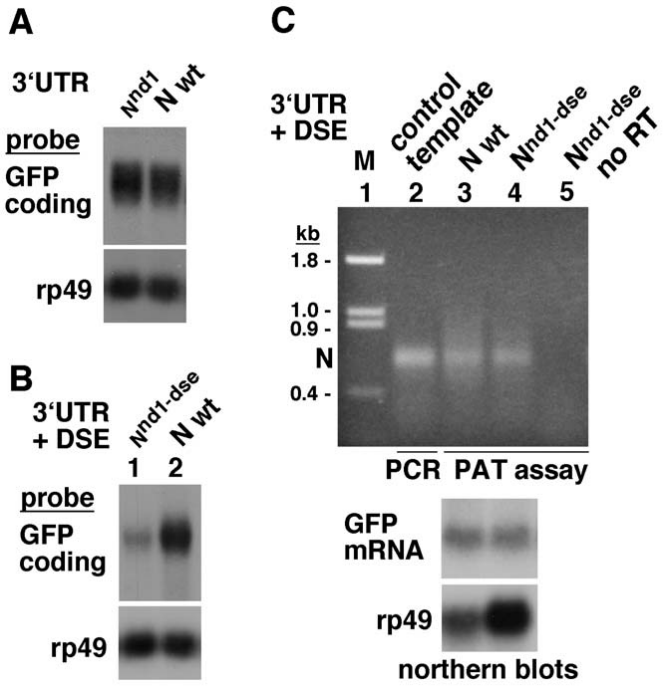
The mutation in the DSE and not polymorphisms or T deletion/insertion in the 3′ UTR of the original *N^nd1^* allele affects mRNA expression in Drosophila S2 cells. (**A**) Northern blots showing that the 3′ UTR of the *N^nd1^* allele and 3′ UTR of the wild type *N* allele produce comparable levels of GFP mRNA. Both constructs contained the actin promoter, GFP coding region, and the wild type N DSE. (**B**) Northern blots showing that addition of the *N^nd1^* DSE mutation to the *N^nd1^* 3′ UTR affects mRNA expression in Drosophila S2 cells. N^nd1-dse^ lane  =  actin promoter +GFP coding+ N^nd1^ 3′ UTR + N^nd1^-dse; N wt lane  =  actin promoter +GFP coding+ N 3′ UTR + N dse. rp49 =  RNA loading control. (**C**) Poly(A) Tail assay (PAT) of RNA samples used in Fig. 2B showing that polyadenylation of residual N^nd1-dse^ mRNA is comparable to the level of polyadenylation of wild type (wt) N mRNA (lanes 3 and 4). A 10∶1 ratio of N^nd1-dse^:wt total RNA was used as it provided comparable levels of N RNA as determined by northern blots (bottom panel). PAT assay was performed using a N specific (5′ primer 2, see Fig. 4D) and an oligo d(T) primer that initiates DNA synthesis all along the length of the poly(A) tail, thereby revealing the level of polyadenylation of N mRNA. Lane 2 is the product of PCR using N cDNA template (control template) and an N primer (3′ primer 3, see Fig. 4D) ending at the N mRNA cleavage site to indicate the size of the fragment without the poly(A) tail. Ethidium bromide gel images are shown. no RT  =  reverse transcriptase omitted for cDNA synthesis.

We sequenced the entire DNA corresponding to the N intracellular domain, the 3′ UTR, and the downstream sequence (∼ 500 bp) in three iso-chromosomal lines with the deletion and three lines without the deletion. In the coding region of both types of lines, we found two polymorphisms that do not alter the amino acid sequence (p1 and p2 in **Fig. 1E**). In the 3′ UTR region of both types of lines we discovered three classes of changes. The first class contained three polymorphisms that are also present in flies with the wild type phenotype (p3, p6, and p7 in Fig. 1E; http://flybase.org). The second class of change was either a T deletion within a run of 15 Ts or a T insertion within a run of 12 Ts in regions that are poorly conserved across the 12 sequenced *Drosophila* species, including changes in the number of Ts (p4 and p5 in Fig. 1E). In Drosophila S2 cells, the wild type N 3′ UTR and the *N^nd-1^* 3′ UTR (with polymorphisms p3 to p7) linked to the actin promoter and the GFP coding sequence produced comparable levels of mRNA (**Fig. 2A**). This result was not surprising as these polymorphisms were too far upstream of the consensus poly(A) site (>290 bp) to affect mRNA 3′ processing. We conclude that the above two classes of changes are not the cause of the *N^nd-1^* phenotype.

**Figure 3:**
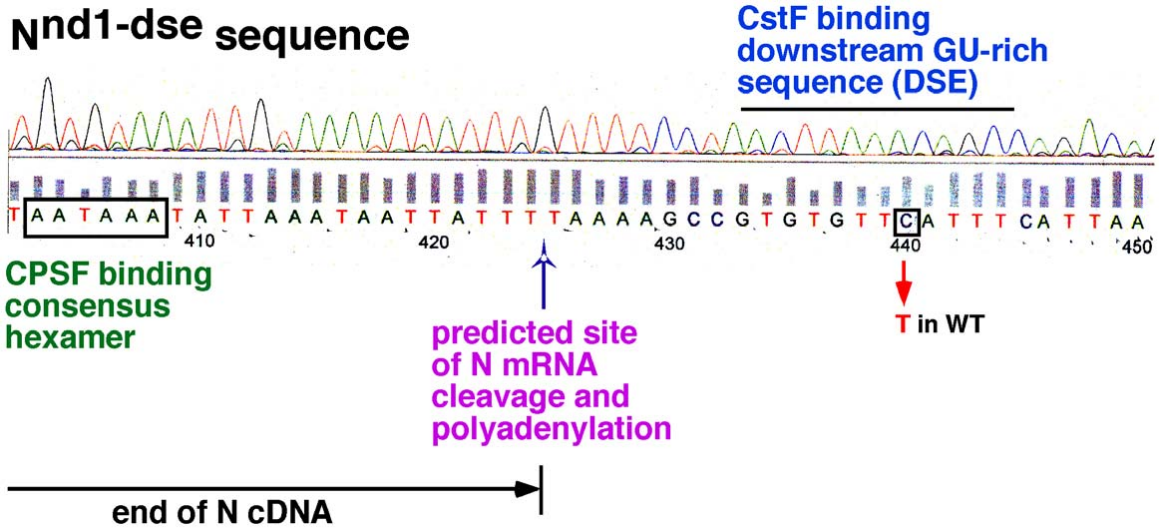
*N^nd1-dse^* mutation is in the GU-rich Down Stream Element (DSE) of the N consensus poly(A) site. Important features of the poly(A) site are marked on the actual sequencing read-out of the *N^nd1-dse^* sequence revealing the site of mutation.

The third class of change was a T > C transition (position 9408 in N cDNA sequence) within the highly conserved GU-rich Downstream Sequence Element (DSE) of the N consensus polyadenylation (poly(A)) site (**Fig. 3**). DSE is the binding site for mRNA 3′ processing factor Cleavage Stimulation Factor (CstF). CstF stimulates the activity of the basic mRNA 3′ processing complex that also contains Cleavage/Polyadenylation Specificity Factor (CPSF) and Poly(A) Polymerase (PAP). This complex binds mRNA in the region encompassing the AAUAAA hexamer and the GU-rich DSE, cleaves the nascent mRNA at a specific site called the cleavage site, and polyadenylates it. Polyadenylation is critical for mRNA stability, nuclear export, and translation [41]–[51]. Thus, the DSE mutation was expected to reduce mRNA levels.

To examine the possibility that the DSE mutation reduces mRNA levels, we cloned N 3′ UTR and downstream sequence, with and without the DSE mutation, after the GFP coding sequence linked to the actin 5C promoter. These constructs were expressed in S2 cells and levels of RNA assessed by northern blotting. Results of these studies confirmed that the DSE mutation suppresses mRNA expression in S2 cells (**Fig. 2B**). To determine if the DSE mutation affected polyadenylation as well, we first determined the ratio of total RNA amount that would contain approximately the same amount of GFP mRNA with and without the DSE mutation. We found it to be about 1∶10 (see the bottom panel in **Fig. 2C**). We used this ratio of RNA in Poly(A) Tail (PAT) assays with a N 3′ UTR specific primer and poly(T) primer that would initiate cDNA synthesis all along the length of the poly(A) tail and reveal any difference in polyadenylation. We found comparable levels of polyadenylation in GFP mRNA with and without the DSE mutation (**Fig. 2C**). These data indicated that the T>C mutation in the *N* DSE is the cause of mutant phenotypes associated with the original N^nd-1^ allele and suggested that it might reduce the stability of unprocessed N pre-mRNA *in vivo* leading to less mature mRNA. An effect on polyadenylation was not expected.

### SAT *N^nd-1^* and DSC *N^nd-1^* Alleles Are Closely Related and Manifest Similar Mutant Phenotypes

Sequences from iso-chromosomal lines with the deletion (isolated from the SAT *N^nd-1^* stock) were identical to the sequence from the DSC *N^nd-1^* stock (which contains the same deletion). In other words, three sets of *N^nd-1^* lines (SAT without deletion, SAT with deletion, and DSC with deletion) can be grouped into two classes: *N^nd-1^* with or without the Harding-Theobald et al. (2007) deletion. These two classes manifest similar morphological and molecular phenotypes (data not shown). As all three sets of *N^nd-1^* lines share the DSE mutation but only two share the deletion mutation, the parsimonious conclusion is that the DSE mutation is the ancestral mutation. Thus, it appears that the deletion reported by Harding-Theobald et al. [40] originated in the SAT *N^nd-1^* fly stock and it is close to displacing the original *N^nd-1^* allele from this stock; the displacement is complete in the DSC *N^nd-1^* stock. From here onwards, we will focus on the ancestral (original) *N^nd-1^* allele, which will be referred to as *N^nd1-dse^*.

### The *N^nd1-dse^* Allele Produces More Polyadenylated N mRNA and N^intra^

**Figure 4:**
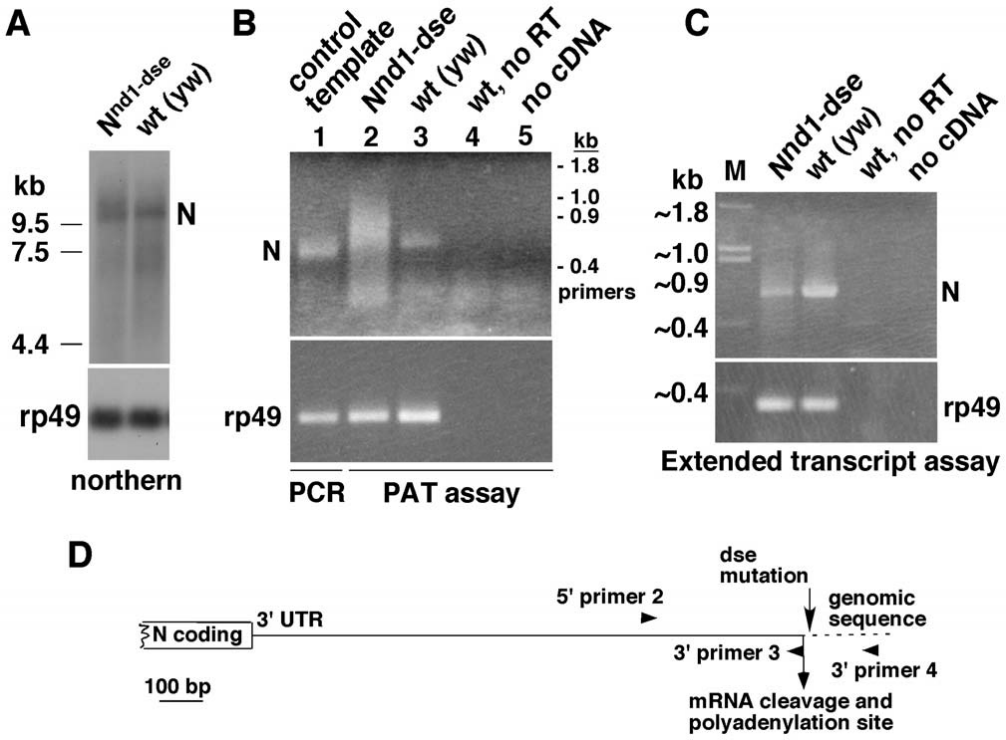
*N^nd1-dse^* embryos produce higher levels of poly(A)-tailed N mRNA and N^intra^. (**A**) Northern blots showing comparable levels of N mRNA in N^nd1-dse^ and wild type embryos after 60 minutes at the restrictive temperature of 30°C. (**B**) PAT assay using N specific (5′ primer 2) or rp49 specific primer and an oligo d(T) primer that reveals the level of polyadenylation of N mRNA (top panel) and the control rp49 mRNA (bottom panel). The smear of fragments of heterogeneous lengths that is present only in the N^nd1-dse^ lane indicates that N mRNA is poly(A)-tailed to a higher level in N^nd1-dse^ embryos than in wild type embryos. Lane 1 is the product of PCR using N cDNA template (control template) and an N primer (3′ primer 3) ending at the N mRNA cleavage site. Ethidium bromide gel images are shown. no RT  =  reverse transcriptase omitted in the cDNA synthesis reaction. rp49 =  rp49 PAT fragments amplified from the same samples that served as controls. (**C**) Unprocessed (extended) transcript assay using one primer upstream of the mRNA cleavage site (5′ primer 2) and one primer downstream of the cleavage site (3′ primer 4). Only N transcripts that bypass the consensus poly(A) site are expected to be amplified. To assess the level of total RNA in the reactions, primers located within the rp49 cDNA were used. Ethidium bromide gel images are shown. **D**. Schematic representation of the primers used for results presented in **B** and **C**.

Northern blotting analysis showed that N mRNA in *N^nd1-dse^* embryos generally ‘smears upwards’ suggesting increased mRNA length due to polyadenylation. A sample northern blot with comparable amounts of N mRNA in *N^nd1-dse^* and wild type (*y, w*) embryos is shown in **Figure 4A**. An increase in the size of mRNA could be due to alteration in mRNA cleavage (the DSE mutation results in a CA sequence doublet that is frequently used for cleavage in the context of a poly(A) site), alteration in polyadenylation (fraction of mRNA polyadenylated or the length of ploy(A) tail), or increase in unprocessed (extended) transcripts. We examined these possibilities with the same RNA samples used in Figure 4A. First, we performed RT-PCR using a N specific 5′ primer and a poly(T) 3′ primer containing a degenerate base (A/G/C) at the 3′ end. The latter primer should initiate synthesis at the base preceding the poly(A) tail, thereby marking the site of mRNA cleavage. Sequencing of these RT-PCR fragments showed that mRNAs from both *N^nd1-dse^* and wild type embryos were cleaved and poly(A) tailed at the predicted cleavage site (data not shown; see Figure 3 for the cleavage site). Next, we performed RT-PCR with an N-specific 5′ primer and a poly(T) 3′ primer, which would hybridize all along the length of the poly(A) tail and reflect the extent of poly(A) tails. Results from these experiments showed increased amounts of poly(A) tailed N mRNA in *N^nd1-dse^* embryos (**Fig. 4B**). The same assay performed on the control rp49 mRNA in the same samples showed low and comparable levels between *N^nd1-dse^* and wild-type embryos (**Fig. 4B**, **bottom panel**). RT-PCR with one primer before the N mRNA cleavage site and one primer downstream of this site showed that the levels of unprocessed (extended) N transcripts were lower in *N^nd1-dse^* embryos, which is consistent with an increased amount of the mature N mRNA in this sample (**Fig. 4C**). Thus, it appears that the DSE mutation increased processing of the N mRNA and reduced bypassing of the N consensus poly(A) site.

### *N^nd1-dse^* Embryos Manifest Gain of N Signaling Phenotypes

**Figure 5:**
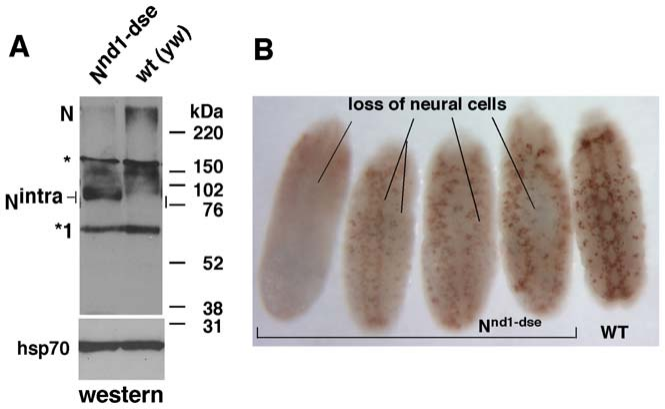
*N^nd1-dse^* embryos show gain-of-N signaling molecular and developmental phenotypes. (**A**) Western blots showing that *N^nd1-dse^* embryos overproduce N^intra^. Statistical analysis of values standardized to the level of hsp70 showed that *N^nd1-dse^* embryos produce 28.8X higher N^intra^ and 9.2X lower full length N compared to wild type embryos (p<0.01 for both, n = 3). N  =  full length N protein; * =  non-specific band; *1 =  the dominant-negative NΔCterm fragment [22], [25]. (**B**) Neuronal cells are lost to varying degrees in *N^nd1-dse^* embryos, as expected with the gain in N signaling. Embryos were probed for the Hunchback protein, a neurogenesis marker.

To determine if an increased level of mature N mRNA affected the production of either the full length N protein or N^intra^, we performed western blotting analysis. *N^nd1-dse^* embryo samples contained a much higher level of N^intra^ compared with the level in wild-type embryos (**Fig. 5A**). The lower level of full length N is possibly a consequence of rapid conversion to N^intra^ and negative regulation by N^intra^ (see discussion). Given the presence of higher amounts of N^intra^, we predicted that *N^nd1-dse^* embryos would manifest gain of N signaling phenotypes. To test this prediction, we studied neurogenesis where N functions are best understood. During neurogenesis, clusters of 12–20 cells first acquire the potential to become neuronal cells. These cells are called proneural cells. N signaling is inhibited in 1–2 proneural cells within each cluster to commit them to the neuronal fate. N signaling is increased in the remaining proneural cells in the cluster to commit them to the alternative epidermal fate. As a consequence, embryos with reduced N signaling manifest excess neuronal cells and embryos with increased N signaling manifest loss of neuronal cells [1], [13], [35]. We found that *N^nd1-dse^* embryos manifest varying degrees of loss of neuronal cells (**Fig. 5B**). Comparison of *N^nd1-dse^* and wild-type embryos at different stages of development indicate that about 50% of each stage of *N^nd1-dse^* embryos manifested syndromes of defects consistent with increased N signaling (data not shown). These data confirmed our prediction from molecular analyses that the *N^nd1-dse^* mutation results in a gain of N signaling. These results suggest that the level of N^intra^ is initially kept low via the suppression of mRNA 3′ processing at the consensus N poly(A) site. This suppression is disrupted by the *N^nd1-dse^* mutation. As a consequence, N signaling is excessive and embryogenesis is severely disrupted.

## Discussion

Our data show that the original N^nd1^ allele, which is designated N^nd1-dse^, is mutated in the DSE of the consensus poly(A) site of the *N* gene. DSE is well known to be required for mRNA 3′ processing and polyadenylation. Thus, a mutation in the N DSE was expected to reduce N mRNA 3′ processing and polyadenylation. We find the contrary result: N mRNA 3′ processing and polyadenylation is increased in N^nd1-dse^ embryos. Accordingly, N signaling is excessive in these embryos and embryogenesis is severely disrupted.

One possible explanation for our unexpected result is that the *N^nd1-dse^* mutation in the DSE increases the activity of one of the component of the basic mRNA 3′ processing complex, for example CstF. If this were the case, we expected to have observed higher levels of mRNA and polyadenylation from DSE mutation constructs in cultured S2 cells as these cells contain all the basic mRNA 3′ processing factors. Instead, we observe a lower level of mRNA and no change in polyadenylation compared to the control construct. The lower level of mRNA from the mutated DSE construct is consistent with the interpretation that unprocessed transcripts are rapidly degraded. An alternative explanation for increased polyadenylation with the DSE mutation is based on the report from mammalian systems that the DSE can act as a binding site for a negative regulator of mRNA 3′ processing and polyadenylation [52]. Thus, poly(A) tailing of N mRNA in embryos might be normally kept low by a negative regulator that is not part of the basic mRNA 3′ processing complex, one that keeps poly(A) tailed N mRNA even lower than the level produced with impaired CstF function. In other words, the DSE mutation in the *N^nd1-dse^* allele might reduce the activity of this negative regulator more than it affects the function of CstF, resulting in a net increase in N mRNA 3′ processing that in turn increases N mRNA polyadenylation and translation, N^intra^ production, and Notch signaling.

The higher level of N^intra^ in *N^nd1-dse^* embryos is consistent with N mRNA polyadenylation being a limiting factor in the production of N^intra^. Interestingly, the level of the full-length N protein (which is the substrate for N^intra^ production) is reduced rather than increased. One possibility is that the DSE mutation somehow affects the N^intra^-producing N proteolysis mechanism operating at the cell surface (or in cytoplasmic vesicles). There is no known mechanism (nor can we imagine one) that links an mRNA 3′ processing mutation in the DSE to proteolysis of a protein at the cell surface or the cytoplasm (note that this mutation which lies outside the cleavage site is not expected to be in the polyadenylated mRNA transported to the cytoplasm for translation). We favor the alternative possibility that the reduction in the level of the full-length N protein in *N^nd1-dse^* embryos is a combined effect of rapid conversion to N^intra^ and suppression due to N^intra^ over-expression. It is well known in the field that although the full length N protein is easily detected in western blots of wild type embryonic extract, N^intra^ is barely detectable indicating that the wild type N expression does not lead to promiscuous N^intra^ production. However, with even a mere 1.5 fold increases in endogenous N expression excess N signaling becomes apparent [11], [12], [14], [15]. In our experiments, we frequently detected mild accumulation of the full length N protein in *N^nd1-dse^* embryos for a brief period (between 15 to 30 minutes) after transfer to the restrictive temperature and before the accumulation of N^intra^ (data not shown). These observations suggest that any increase in the level of the full-length N protein beyond the wild type level tips the balance towards increased processing of N to generate N^intra^. Our previous studies have shown that increased N^intra^ production from a transgenic construct (that directly produces this molecule) suppresses the expression of the full length N protein from the endogenous gene in the background [22], [35], [36]. Thus, the loss of full length N protein in N^nd1-dse^ embryos could be a consequence of increased N^intra^ production.

We interpret our data as indicating that N mRNA 3′ processing and polyadenylation is subjected to strong negative regulation. This interpretation is supported by two well known and long-standing observations: (1) sensitivity of development to a mere 1.5–2X difference in *N* gene dosage or very small differences in the level of N signaling and (2) low, uniform expression of the Notch protein throughout the embryo [10]–[20]. The negative regulator could be one of the RNA binding proteins identified in genetic screens as a suppressor of N signaling [27], [28]. However, given the temperature sensitivity of the *N^nd1-dse^* allele [34], it could also be the local structure of the N mRNA region encompassing the DSE. At this time, the mechanism is obscure but all indications are that it is unusual and novel, one that doesn't fit the known aspects of mRNA 3′ processing. The primary function of the N DSE appears to be prevention of mRNA 3′ processing rather than its promotion, which would explain the bypass of the consensus poly(A) site and production of extended transcripts. This mRNA 3′ processing mechanism could be an important regulator of N expression. The Notch promoter in Drosophila has remained elusive despite intense efforts by many laboratories and it appears that none of the many protein level regulatory mechanisms was able to check the abnormal molecular and morphological phenotypic effects of the N^nd1-dse^ mutation.

In summary, N is a basic regulator of development in all animals and a small difference in its function is frequently used during development to specify alternative cell fates, establish boundaries between tissues, or refine morphological patterns. Small perturbations in N activity lead to numerous human developmental defects and diseases including cancer, stroke, and dementia. As N activity is based on N^intra^, our data showing that the loss of N DSE function results in a high level of N^intra^ production suggests that the mechanism regulating N mRNA 3′ processing is a critical regulator of N signaling. This mechanism appears to use mRNA 3′ processing elements for default suppression of N mRNA processing at the consensus poly(A) site. Our data and the N^nd1-dse^ embryo could be very useful for further studies aimed at dissecting this mechanism. They might also be useful for studies aimed at understanding how the many RNA binding proteins identified in genetic screens fit into the regulation of N signaling.
